# Prevalence of Lewy body pathology and phenotypic associations in patients with mild cognitive impairment: Evidence from the Interceptor study

**DOI:** 10.1002/dad2.70382

**Published:** 2026-06-15

**Authors:** Simone Baiardi, Serena Caldera, Sabina Capellari, Franco Magliocchetti, Angela Mammana, Corrado Zenesini, Stefano Cappa, Naike Caraglia, Maria Cotelli, Camillo Marra, Francesca Miraglia, Daniela Perani, Alberto Redolfi, Patrizia Spadin, Fabrizio Tagliavini, Nicola Vanacore, Fabrizio Vecchio, Paolo Maria Rossini, Piero Parchi

**Affiliations:** ^1^ Department of Biomedical and Neuromotor Sciences University of Bologna Bologna Italy; ^2^ IRCCS Istituto delle Scienze Neurologiche di Bologna Bologna Italy; ^3^ University Institute of Advanced Studies IUSS Pavia Italy; ^4^ IRCCS Istituto Auxologico Milan Italy; ^5^ Memory Clinic, Fondazione Policlinico Universitario “A. Gemelli” IRCCS Rome Italy; ^6^ IRCCS Istituto Centro San Giovanni di Dio Fatebenefratelli Brescia Italy; ^7^ Department of Psychology Catholic University of Sacred Heart Milan Italy; ^8^ Department of Theoretical and Applied Sciences eCampus University Novedrate Italy; ^9^ Brain Connectivity Laboratory Department of Neuroscience and Neurorehabilitation IRCCS San Raffaele Roma Rome Italy; ^10^ Vita‐Salute San Raffaele University Milan Italy; ^11^ Associazione italiana Malattia di Alzheimer, AIMA Milan Italy; ^12^ Fondazione IRCCS Istituto Neurologico Carlo Besta Milan Italy; ^13^ National Center for Disease Prevention and Health Promotion National Institute of Health Rome Italy; ^14^ Department of Neuroscience and Neurorehabilitation IRCCS San Raffaele Roma Rome Italy

**Keywords:** alpha‐synuclein, Alzheimer's disease, dementia with Lewy bodies, real‐time quaking‐induced conversion assay

## Abstract

**INTRODUCTION:**

Clinical effects and prevalence of Lewy body (LB) pathology in patients with mild cognitive impairment (MCI) remain poorly understood.

**METHODS:**

We assessed LB pathology in 339 patients with MCI from the Italian multicenter Interceptor cohort using the cerebrospinal fluid α‐synuclein seed amplification assay. Baseline assessment was followed by serial cognitive evaluations over 3 years.

**RESULTS:**

LB+ participants (*n *= 50, 15%) were older and showed a higher frequency of parkinsonism, falls, and delirium episodes than those LB–, although these differences were not statistically significant. At follow‐up, patients with biomarker evidence of Alzheimer's disease (AD), either isolated (AD+/LB–) or with concomitant LB pathology (AD+/LB+), showed the fastest cognitive decline and progression to dementia.

**DISCUSSION:**

In a multicenter MCI cohort, LB pathology was associated with older age and an increased frequency of motor/psychiatric symptoms. Larger studies are needed to clarify the independent contribution of AD and LB pathologies to the MCI phenotype and clinical progression.

## Highlights

- A cerebrospinal fluid α‐synuclein seed amplification assay detects Lewy body (LB) pathology in 15% of mild cognitive impairment (MCI) patients.
- LB pathology is associated with older age and increased absolute frequency of motor/psychiatric symptoms.
- Co‐existence of LB and Alzheimer's disease (AD) pathologies seems to promote a faster global cognitive decline.
- α‐synuclein seed amplification assay is an added value in the diagnostic workup of MCI patients.
- Detection of LB comorbidity is indicated for the proper assessment of anti‐amyloid treatment efficacy in patients with AD.

## 1 BACKGROUND

The seed amplification assays for misfolded alpha‐synuclein (αSyn SAA) have provided a reliable biomarker for Lewy body (LB) pathology. Previous studies have shown that αSyn SAA accurately detects LB pathology in patients at the mild cognitive impairment (MCI) stage, either as a primary pathology or associated with Alzheimer's disease (AD).^1^, ^2^, ^3^, ^4^ We recently identified LB pathology in 17% of 481 individuals with MCI from the BioFINDER‐1 and ‐2 cohorts.^2^ In the same study, patients with LB pathology showed worse performance in attention/executive, visuospatial, and motor functions, and a higher prevalence of visual hallucinations, independent of amyloid beta (Aβ) and tau pathologies. Moreover, independent studies demonstrated that αSyn SAA positivity in cognitively impaired individuals predicts a faster longitudinal decline in cognitive function.^2^, ^4^, ^5^ To expand on this initial evidence, we used a biomarker‐driven approach to assess the prevalence of LB pathology and its effect on clinical and laboratory phenotypes in a multicenter cohort of Italian patients diagnosed with MCI.

## 2 METHODS

### 2.1 Study design and participants

In this prospective study, we used a purely biomarker‐driven classification of AD pathology (AD+), defined by the concomitant positivity of cerebrospinal fluid (CSF) Aβ and tau markers (see below), and of LB pathology (LB+), defined by CSF αSyn SAA positivity. A total of 339 patients from the Italian Interceptor cohort were included.^6^ The Interceptor project is a longitudinal, multicenter, non‐therapeutic cohort study including patients diagnosed with MCI (https://www.interceptorproject.com/en/). The core criteria for MCI diagnosis were defined according to the National Institute on Aging–Alzheimer's Association (NIA‐AA) workgroups.^7^ Participants were recruited by 19 centers with a documented expertise in diagnosis and treatment of MCI and AD (Centri per i Disturbi Cognitivi e Demenza [CDCD]) distributed across the Italian national territory. Inclusion criteria were the following: (1) age between 50 and 85 years; (2) age‐ and education‐corrected Mini‐Mental State Examination (MMSE) ≥ 24; (3) Clinical Dementia Rating (CDR) global score of 0.5; (4) concerns about cognitive modifications, expressed as subjective complaints by the subject, or by impression by a close acquaintance or an expert clinician; (5) defective performance with reference to age‐ and education‐matched controls in one or more cognitive domain(s); and (6) preserved functional autonomy. Exclusion criteria were ahistory of cerebrovascular disease, alcohol abuse, comorbidities affecting cognition (e.g., thyroid disease), human immunodeficiency virus infection, neuroimaging evidence of other potential causes of cognitive decline (e.g., subdural hematoma, malignancy), chronic treatment with psychotropic drugs, history of malignancy ≤ 5 years, women in reproductive age, contraindications for magnetic resonance imaging (MRI) or lumbar puncture, and participation in trials with an experimental drug. Each subject proposed for enrollment by each recruiting center was then confirmed by two senior neurologists from the study's scientific committee.

The study was approved by the ethics committee of Fondazione Policlinico Universitario Agostino Gemelli (approval code 2251) and then by all local ethics committees of the participating clinical centers. Written informed consent was obtained from all subjects prior to study participation.

### 2.2 Baseline assessment

At baseline, participants underwent a detailed sociodemographic and clinical interview, neurological examination, neuropsychological testing (Cognitive Function Instrument^8^ for subjective complaints; MMSE^9^ and CDR^10^ for global cognitive functioning; Free and Cued Selective Reminding Test [FCSRT],^11^ Rey Auditory Verbal Learning Test [RAVLT],^12^ delayed recall of Rey Osterrieth Complex Figure [ROCF],^13^ and Episodic Memory Score^14^ for both verbal and visuo‐spatial memory; Screening for Aphasia in Neurodegeneration^15^ and phonemic and semantic verbal fluency^16^ for language; Poppelreuter–Ghent overlapping figures^17^ and copy task of ROCF for visuo‐spatial function; Trail Making Test,^18^ Stroop color and word test,^19^ Raven's Coloured Progressive Matrices,^20^ and Frontal Assessment Battery^21^ for executive function; Neuropsychiatric Inventory^22^ for behavioral disturbances; and Amsterdam IADL Questionnaire—short version^23^ for functional assessment), brain [18F]fluorodeoxyglucose positron emission tomography (PET), structural MRI for hippocampal volumetry, apolipoprotein E (*APOE*) genotyping, and CSF collection by lumbar puncture for determination of CSF AD core biomarkers (phosphorylated tau [p‐tau]181, total tau [t‐tau], Aβ42/Aβ40).

**RESEARCH IN CONTEXT**

1. **Systematic Review**: Cerebrospinal fluid α‐synuclein seed amplification assays accurately detect Lewy body disease at the mild cognitive impairment stage. Cohort studies have provided initial evidence of the effects of Lewy body (LB) pathology on clinical manifestations and longitudinal progression of cognitive decline, but confirmatory data are lacking.
2. **Interpretation**: In a multicenter cohort of patients with mild cognitive decline, we confirmed a notable prevalence (15%) of LB pathology and a trend toward an accelerated cognitive worsening in patients with comorbid Alzheimer's disease (AD) and LB diseases compared to AD alone. These results provide further supporting evidence for the use of the α‐synuclein seed amplification assay in the diagnostic workup of patients with cognitive decline.
3. **Future Directions**: In the era of disease‐modifying treatments for AD, the accurate detection of LB comorbidity will represent a significant step toward precision medicine, also contributing to a more accurate assessment of candidates for anti‐amyloid efficacy in clinical trials.

### 2.3 CSF biomarker analyses

CSF p‐tau181, t‐tau, and Aβ42/Aβ40 were analyzed by fully automated chemiluminescence enzyme immunoassay on the Lumipulse G600II platform (Fujirebio).^24^ CSF αSyn SAA was performed at IRCCS Istituto delle Scienze Neurologiche di Bologna according to our previously published protocol.^25^ Briefly, six 0.8‐mm silica beads (OPS Diagnostics) per well were pre‐loaded into black 96‐well plates with a clear bottom (Nalgene Nunc International). CSF samples were thawed and vortexed for 10 seconds before use. Then, 15 µl CSF was added to 85 µl reaction mix composed of 40 mM PB, pH 8.0, 170 mM NaCl, 10 mM thioflavin‐T (Sigma), 0.0015% SDS (Bio‐Rad), and 0.1 g l−1 of filtered recombinant αSyn (100‐kDa Amicon centrifugal filters, Merck Millipore). Plates were closed with a plate sealer film (Nalgene Nunc International) and incubated in a FLUOstar Omega plate reader (BMG Labtech) at 42°C with intermittent double orbital shaking at 400 rpm for 1 minute, followed by 1 minute rest. Fluorescence was measured every 45 minutes with 450 nm excitation and 480 nm emission filters during the 30‐hour test run. Samples and controls were run in quadruplicate and considered positive after the first run if at least three out of four replicates exceeded a threshold of 30% of the median Imax values of the positive control replicates. To minimize the risk of false‐positive results, we repeated the analysis of samples with one or two positive replicates in the first run three times. We considered a positive result only when at least 4 of the 12 total replicates reached the threshold.

The CSF Aβ42/Aβ40 ratio (cut‐off ≤ 0.06821)^26^ was used to define Aβ positivity, whereas tau positivity was defined as abnormal CSF p‐tau181 (cut‐off ≥ 56 pg/ml).^27^ The αSyn SAA result was used to define LB positive/negative (±) status.

### 2.4 Longitudinal assessment

Participants underwent serial clinical/neuropsychological evaluations every 6 months over a 3‐year follow‐up period. MMSE and CDR scores, as well as conversion to dementia, were assessed at each follow‐up visit. The conversion to dementia was diagnosed according to Diagnostic and Statistical Manual of Mental Disorders Fifth Edition based on clinical, cognitive, and functional examination. Longitudinal follow‐up data (at least one evaluation) were available in 323 subjects.

### 2.5 Statistical analyses

The normality of continuous variables was assessed using Shapiro–Wilk and Kolmogorov–Smirnov tests. For group comparisons, one‐way analysis of variance (followed by a Tukey post hoc test) was used to analyze normally distributed variables, while the Kruskal–Wallis test (followed by a Dunn post hoc test) was used for non‐normally distributed continuous variables. Categorical variables were compared using chi‐squared or Fisher exact test as appropriate. In cross‐sectional analyses, general linear regression models were used to test differences in cognitive scores (dependent variables) between AD/LB groups (independent variables), adjusting for age and sex. Next, binarized Aβ, tau, and LB pathology (to facilitate more straightforward comparison of estimates) were used instead of the AD/LB group. Logistic regression models were used for the binary clinical outcomes (presence of hallucinations, parkinsonism, fluctuation, and delirium episodes). The Kaplan–Meier method was used to estimate the cumulative, time‐dependent probability of conversion to dementia. Multivariable Cox proportional hazards regression models were then used to evaluate the association between time to conversion and the combined AD/LB group, adjusting for age (at each evaluation) and sex. Results are presented as hazard ratios (HRs) with corresponding 95% confidence intervals (CIs). Linear mixed‐effects models with random intercepts and slopes were used to assess the longitudinal changes in MMSE and CDR scores. Separate models were fitted for each dependent variable, with time (in months) as the independent variable, adjusting for age (at each evaluation) and sex. To evaluate whether the slope of change over time differed by the combined AD/LB group, an interaction term between time and group was included. The significance of the interaction was assessed using a likelihood ratio test comparing models with and without the interaction term. When the interaction was significant, group‐specific longitudinal trajectories were estimated from the model and presented for each of the four groups. Estimated effects are reported as beta coefficients (β) with 95% CI. A two‐sided *p* value < 0.05 was considered to indicate statistical significance. The statistical analyses were performed using GraphPad and STATA software.

## 3 RESULTS

Participants had a mean age of 71.7 ± 7.0 years, 50.1% were male, 11.2% were Aβ+/tau–, and 46.0% Aβ+/tau+. The αSyn SAA showed positive seeding activity in 50 (14.7%) participants (Table 1); 33 of them (66.0%) were Aβ+, and 23 (46.0%) were Aβ+/tau+. The AD+ group was enriched for *APOE* ε4 carriers with no differences between AD+/LB– and AD+/LB+.

**TABLE 1:** Main clinical and demographic characteristics of AD/LB groups.

	AD–/LB– (*n* = 156)	AD–/LB+ (*n* = 27)	AD+/LB– (*n* = 133)	AD+/LB+ (*n* = 23)	*p* value	Total (*n* = 339)
Age, years	69.9 (7.9)	74.2 (4.1)	72.9 (5.9)	74.4 (5.7)	0.023^§^, 0.003^#^, 0.012^‡^	71.7 (7.0)
Sex, *n* females	72 (46.2%)	5 (18.5%)	77 (58.3%)	15 (65.2%)	0.01^§^, 0.045^#^, < 0.001^¥^, 0.001^¶^	169 (49.9%)
Presenting symptom(s), *n*:					
Memory loss Language disturbance Attention deficit Disorientation	149 (96.8%) 29 (18.8%) 55 (35.5%) 30 (19.2%)	26 (96.3%) 1 (3.7%) 13 (48.1%) 3 (11.1%)	128 (96.2%) 14 (10.7%) 30 (22.9%) 26 (19.5%)	23 (100%) 2 (8.7%) 5 (21.7%) 4 (17.4%)	0.777 0.082 0.027^#^, 0.016^¥^ 0.821	326 (96.7%) 46 (13.7%) 103 (30.7%) 63 (18.6%)
Hallucinations, *n*	0 (0.0%)	1 (3.7%)	0 (0.0%)	0 (0.0%)	0.148	1 (0.3%)
Fluctuations, *n*	4 (2.5%)	2 (7.4%)	1 (0.8%)	0 (0.0%)	0.144	7 (2.1%)
Parkinsonism, *n*	3 (1.9%)	2 (7.4%)	0 (0.0%)	1 (4.2%)	0.027^¥^	6 (1.8%)
Delirium episodes, n	1 (0.6%)	2 (7.4%)	2 (1.5%)	0 (0.0%)	0.119	5 (1.5%)
Falls, *n*	7 (4.5%)	3 (11.1%)	5 (3.8%)	3 (13.0%)	0.106	18 (5.3%)
*APOE* ε4 (at least one allele), *n*	35 (23.3%)	6 (22.2%)	79 (60.3%)	15 (68.2%)	<0.001^#,‡,¥^, 0.002^¶^	135 (40.9%)
CSF total tau, pg/ml	281.2 (164.2)	269.9 (201.9)	712.3 (314.2)	655.7 (269.7)	<0.001^#,‡,¥,¶^	475.4 (322.5)
CSF p‐tau181, pg/ml	38.5 (23.5)	34.7 (12.5)	114.5 (53.1)	110.8 (60.5)	<0.001^#,‡,¥,¶^	72.9 (55.2)
CSF Aβ42/Aβ40	0.090 (0.024)	0.082 (0.022)	0.044 (0.010)	0.042 (0.007)	<0.001^#,‡,¥,¶^	0.068 (0.029)
CSF Aβ42/p‐tau181	26.9 (12.7)	23.8 (11.3)	5.5 (2.8)	4.7 (2.0)	<0.001^#,‡,¥,¶^	16.8 (14.1)
Volume right hippocampus, mm^3^	3389 (576)	3152 (383)	3107 (499)	3073 (519)	<0.001^#^	3235 (545)
Volume left hippocampus, mm^3^	3260 (538)	3097 (392)	2994 (474)	2904 (384)	<0.001^#,‡^	3117 (510)
Hypometabolism [18F]FDG PET, *n*	112 (73.7%)	20 (74.1%)	110 (87.3%)	20 (90.9%)	0.006^#^	262 (80.1%)
AD‐like hypometabolism pattern, n^*^	36 (32.1%)	9 (45.0%)	74 (67.3%)	14 (70.0%)	<0.001^#^, 0.002^‡^	133 (50.8%)
Converters to dementia, n^**^	29 (19.6%)	5 (18.5%)	53 (41.7%)	10 (47.6%)	<0.001^#^, 0.004^‡^, 0.028^¥^	97 (30.0%)
Time to conversion, months	18.1 (7.4)	13.6 (6.1)	16.4 (4.5)	12.3 (8.8)	0.156	16.3 (7.6)

*Note*: Data are shown as mean (standard deviation) unless otherwise specified. ^*^The topographic hypometabolism pattern was analyzed in 262 cases showing abnormal metabolism at [18F]FDG PET. ^**^Longitudinal follow‐up data were available in 323 participants (AD–/LB– *n* = 148; AD–/LB+ *n* = 27; AD+/LB– *n* = 127; AD+/LB+ *n* = 21). Demographic factors and clinical characteristics were compared using chi‐squared and Kruskal–Wallis tests: ^§^ AD–/LB– vs. AD–/LB+, ^#^ AD–/LB– vs. AD+/LB–, ^‡^ AD–/LB– vs. AD+/LB+, ^¥^ AD–/LB+ vs. AD+/LB–, ^¶^ AD–/LB+ vs. AD+/LB+, ^†^ AD+/LB– vs. AD+/LB+.

Abbreviations: [18F]FDG PET, [18F]Fluorodeoxyglucose positron emission tomography; Aβ, amyloid beta; AD, Alzheimer's disease; *APOE*, apolipoprotein E; CSF, cerebrospinal fluid; LB, Lewy body; p‐tau, phosphorylated tau.

**FIGURE 1:**
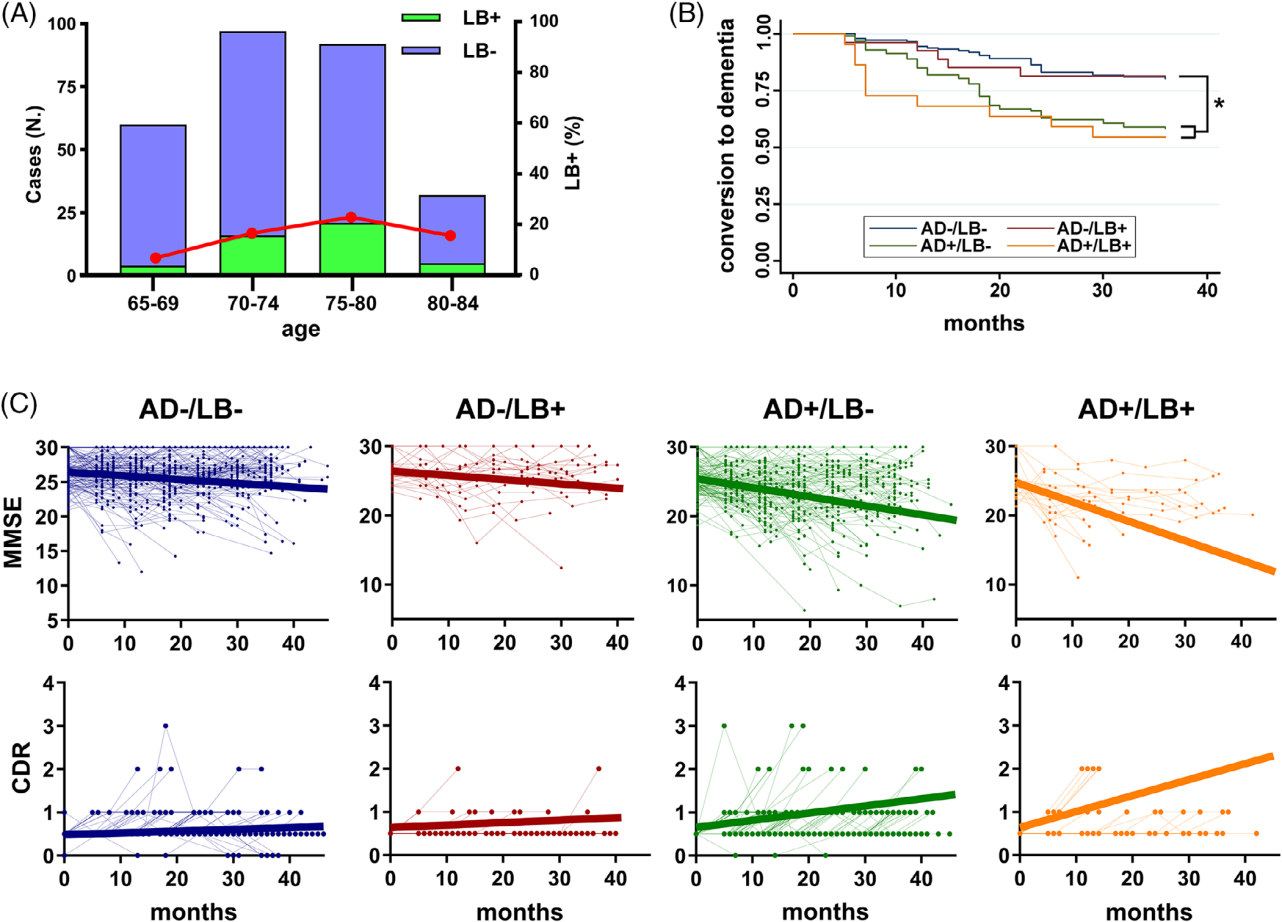
Age‐specific LB pathology prevalence rate in the Interceptor cohort and comparisons between AD/LB groups on longitudinal cognitive function. A, The green area represents the number of positive cases at αSyn SAA in each 5‐year period, starting from age 65, while the red line expresses these data as a percentage. B, Predictive value of AD/LB status in the conversion from MCI to dementia. C, Longitudinal variation of MMSE and CDR scores in the AD/LB groups. Thin dotted lines represent the longitudinal variation at a single‐patient level; thick lines represent the overall parameter longitudinal trend. Missing data are shown in Table S1 in supporting information. αSyn SAA, alpha‐synuclein seed amplification assay; AD, Alzheimer's disease; CDR, Clinical Dementia Rating; LB, Lewy body; MCI, mild cognitive impairment; MMSE, Mini‐Mental State Examination.

LB+ individuals were older compared to those LB–, independent of AD status, with an increasing prevalence after the age of 65 (Figure 1A). Males were overrepresented in the AD–/LB+ group (81.5%), whereas females predominated in the AD+/LB+ group (65.2%; Table 1).

At clinical baseline, LB+ participants showed more frequent attention deficits (36.0% vs. 29.7%) and fewer speech complaints (6.0% vs. 15.1%) than those LB–; however, these differences did not reach statistical significance.

The investigated core clinical features of dementia with Lewy bodies (DLB),^28^ including parkinsonism, fluctuations, and visual hallucinations, as well as medical history relevant for delirium episodes and falls, were infrequent, but more commonly reported in AD–/LB+ (and AD+/LB+, limited to falls) participants compared to those AD–/LB– and AD+/LB–. In logistic regression models, after adjusting for age and sex, LB pathology showed an effect only on parkinsonism (OR 6.1, 95% CI 1.1–33.6, *p* = 0.038) and falls (OR 3.1, 95% CI 1.1–8.7, *p* = 0.037).

The neuropsychological profile was comparable between the AD–/LB– and AD–/LB+ groups, whereas AD+/LB– and AD+/LB+ individuals showed worse performance on memory tests (FCSRT, RAVLT, and ROCF) and category fluency (Table 2). After adjusting for age and sex, the analysis of the independent effect of Aβ, tau, and LB pathologies demonstrated an association of both tau and Aβ with lower scores at FCSRT immediate free recall (tau+: β –0.281, *p *< 0.001; Aβ+: β –0.167, *p *< 0.02), while tau pathology alone correlated with worse results at FCSRT delayed free recall (β –0.308, *p *< 0.001), RAVTL immediate (β –0.226, *p* = 0.002) and delayed recall (β –0.240, *p* = 0.001), and ROCF recall (β –0.219, *p* = 0.003).

**TABLE 2:** Neuropsychological profiles of AD/LB groups.

	AD–/LB– (*n* = 156)	AD–/LB+ (*n* = 27)	AD+/LB– (*n* = 133)	AD+/LB+ (*n* = 23)	*p* value	Total (*n* = 339)
MCI classification:						
amnestic single domain amnestic multiple domains non‐amnestic single domain non‐amnestic multiple domains	69 (44.2%) 64 (51.0%) 14 (8.9%) 9 (5.8%)	14 (51.9%) 10 (37.0%) 2 (7.4%) 1 (3.7%)	79 (59.4%) 48 (36.1%) 2 (1.5%) 4 (3.0%)	9 (39.1%) 11 (47.8%) 3 (13.0%) 0 (0.0%)	0.01^#^ 0.667 0.008^#^ 0.627	171 (50.4%) 133 (39.2%) 21 (6.2%) 14 (4.1%)
MMSE, points	26.5 (2.2)	26.5 (1.8)	25.5 (2.3)	25.3 (2.5)	0.001^#^	26.0 (2.3)
Neuropsychiatric inventory, points	9.8 (10.6)	10.4 (8.6)	8.8 (9.0)	9.3 (9.7)	0.699	9.5 (9.8)
FCSRT IFR, points	23.5 (25.4)	20.3 (6.7)	15.0 (7.2)	12.9 (6.4)	<0.001^#,‡^, 0.011^¥^, 0.005^¶^	18.3 (8.2)
FCSRT ITR raw, points	33.0 (4.8)	32.6 (5.4)	29.1 (6.7)	26.5 (6.9)	<0.001^#,‡^, 0.018^¥^, 0.002^¶^	31.0 (6.2)
FCSRT DFR, points	6.4 (3.9)	6.2 (2.9)	3.6 (3.3)	2.1 (2.9)	<0.001^#,‡,¶^, 0.012^¥^	5.1 (3.8)
FCSRT DTR raw, points	10.6 (2.2)	10.8 (1.9)	8.7 (3.2)	7.9 (2.9)	<0.001^#,‡,¶^, 0.002^¥^	9.7 (2.8)
RAVTL Immediate Recall, points	37.3 (10.1)	38.0 (7.8)	32.5 (8.9)	29.1 (5.9)	<0.001^#,‡^, 0.021^¥^, 0.002^¶^	34.9 (8.6)
RAVTL Delayed Recall, points	6.2 (3.9)	7.1 (3.6)	4.7 (3.3)	2.5 (2.6)	<0.001^‡,¶^, 0.004^#^, 0.019^¥^, 0.038^†^	5.5 (3.7)
Letter Fluency, points	30.2 (9.5)	31.2 (12.2)	31.4 (9.4)	30.0 (9.1)	0.663	30.7 (9.6)
Category Fluency, points	34.9 (9.7)	36.6 (7.7)	31.5 (8.9)	30.1 (7.7)	0.009^#^, 0.021^¥^	33.5 (9.3)
ROCF Copy, points	30.2 (5.7)	30.8 (6.2)	29.7 (6.0)	29.3 (7.4)	0.507	29.9 (5.9)
ROCF Recall, points	13.6 (6.4)	13.3 (7.2)	10.5 (5.9)	7.6 (5.6)	<0.001^#,‡^, 0.012^¶^	11.9 (6.5)
Trial Making Test, seconds:						
Part A Part B Part B‐A	46.8 (33.9) 142.0 (89.7) 98.6 (79.7)	46.9 (33.5) 132.3 (108.5) 87.9 (86.5)	54.8 (49.6) 169.3 (104.0) 118.2 (92.4)	48.2 (33.3) 168.2 (74.7) 121.2 (66.4)	0.547 0.069 0.072	50.1 (40.8) 153.5 (97.0) 106.7 (85.1)
Stroop Color Word test:						
Time, seconds Errors, *n*	24.9 (20.3) 2.1 (5.1)	26.4 (24.8) 1.3 (2.4)	30.3 (51.3) 1.9 (4.2)	30.5 (23.8) 1.4 (2.6)	0.742 0.940	27.5 (36.1) 1.9 (4.4)
Raven's Matrices, points	28.9 (5.4)	29.9 (5.4)	29.2 (4.8)	28.6 (5.6)	0.801	29.1 (5.2)
PGT, points:						
Total Meaningless patterns Meaningful patterns	63.9 (8.1) 30.0 (5.5) 33.8 (3.2)	61.7 (9.9) 28.3 (6.6) 33.4 (3.9)	62.8 (8.6) 29.5 (5.3) 33.2 (3.6)	60.0 (12.2) 27.9 (8.2) 31.9 (6.4)	0.402 0.415 0.398	63.0 (9.0) 29.5 (5.8) 33.4 (3.7)
Frontal Assessment Battery, points	15.6 (2.6)	14.5 (2.3)	13.9 (3.2)	13.6 (3.0)	0.364	14.3 (2.8)

*Notes*: Scores were corrected for age and education years, unless otherwise stated, and compared using Kruskal–Wallis test: ^§^ AD–/LB– vs. AD–/LB+, ^#^ AD–/LB–‐ vs. AD+/LB–, ^‡^ AD–/LB– vs. AD+/LB+, ^¥^ AD–/LB+ vs. AD+/LB–, ^¶^ AD–/LB+ vs. AD+/LB+, ^†^ AD+/LB– vs. AD+/LB+.

Abbreviations: AD, Alzheimer's disease; DFR, Delayed Free Recall; DTR, Delayed Total Recall; FCSRT, Free and Cued Selective Reminding Test; IFR, Immediate Free Recall; ITR, Immediate Total Recall; LB Lewy bodies; MCI, mild cognitive impairment; MMSE, Mini‐Mental State Examination; PGT, Poppelreuter–Ghent's Overlapping Figures Test; RAVTL, Rey Auditory Verbal Learning Test; ROCF, Rey–Osterrieth Complex Figure.

As expected, AD+/LB– and AD+/LB+ participants had a greater hippocampal atrophy rate and a higher frequency of hypometabolism of posterior cingulate cortex, precuneus, and temporoparietal regions (typical AD‐related pattern) at brain [18F]fluorodeoxyglucose PET (Table 1).

In summary, cross‐sectional analyses of LB pathology indicate a subtle overall effect on clinical phenotype, except for a higher prevalence of motor impairment. In contrast, AD+ individuals had greater memory impairment, hippocampal volume reduction, and typical temporo‐parietal hypometabolism.

At longitudinal follow‐up, AD+/LB– and AD+/LB+ groups showed a faster worsening of global cognitive performance than AD–/LB– and AD–/LB+ groups and, accordingly, the highest rate of conversion to overt dementia (AD+/LB–: HR 2.10, 95% CI 1.32–3.32, *p* = 0.002; AD+/LB+: HR 2.55, 95% CI 1.24–5.25, *p* = 0.011; Figure 1B, Table 3). There was a significant difference between groups in the longitudinal worsening of cognitive scores (MMSE, *p* < 0.001, CDR, *p* < 0.001). Focusing on the AD+ groups, AD+/LB+ participants exhibited a greater increase in CDR scores (*p* = 0.007) and, although not statistically significant, a steeper decline in longitudinal MMSE performance (*p* = 0.061) compared to those AD+/LB– (Figure 1C, Table 3). Notably, 8 out of 10 AD+ participants who progressed to dementia within 1.5 years after baseline belonged to the AD+/LB+ group.

**TABLE 3:** Effect of AD/LB status on longitudinal cognitive performance.

Parameter	Group	β coefficient	95% confidence interval	*p* value
**Group‐specific longitudinal trajectories from the mixed effect models**				
MMSE	AD–/LB–	−0.053	−0.072 to –0.035	<0.001
	AD–/LB+	−0.061	−0.102 to –0.021	0.003
	AD+/LB–	−0.131	−0.157 to –0.104	<0.001
	AD+/LB+	−0.281	−0.422 to –0.140	<0.001
CDR	AD–/LB–	0.004	0.002 to 0.006	<0.001
	AD–/LB+	0.005	0.002 to 0.009	0.006
	AD+/LB–	0.016	0.012 to 0.021	<0.001
	AD+/LB+	0.037	0.017 to 0.057	<0.001
**Comparison of longitudinal trajectories: time‐by‐group interactions effects**				
MMSE	AD–/LB–	0.074	0.026 to 0.122	0.002
	AD–/LB+	0.071	−0.011 to 0.153	0.089
	AD+/LB–	reference group		
	AD+/LB+	−0.098	−0.201 to 0.005	0.061
CDR	AD–/LB–	−0.010	−0.015 to –0.005	<0.001
	AD–/LB+	−0.009	−0.018 to –0.001	0.022
	AD+/LB–	reference group		
	AD+/LB+	0.144	0.004 to 0.025	0.007

*Notes*: Significant effects were examined using linear mixed‐effects models. The AD/LB group × time (months) interaction was examined, adjusted for age, sex, and education.

Abbreviations: AD, Alzheimer's disease; CDR, Clinical Dementia Rating; LB, Lewy bodies; MMSE, Mini‐Mental State Examination.

In summary, AD+ participants showed the fastest cognitive decline in longitudinal analyses, with only a trend toward greater cognitive worsening in the AD+/LB+ group relative to the AD+/LB− group.

## 4 DISCUSSION

The early identification of neurodegenerative pathologies underlying cognitive impairment is pivotal for proper clinical management and tailored treatments. We previously demonstrated the high diagnostic value of CSF αSyn SAA in identifying LB pathology at the MCI stage.^1^ In the present study, 15% of Italian patients with MCI in the Interceptor cohort tested positive by CSF αSyn SAA, which is slightly lower than the previously reported rates of 17% to 19% in unselected MCI cohorts,^2^, ^4^ and the 15% to 26% detected in MCI‐AD.^1^, ^3^ In line with the results from the BioFINDER cohorts, AD pathology was found in approximately half of LB+ participants, with a similar prevalence in the AD–/LB+ and AD+/LB+ groups.^2^ Different from BioFINDER, however, the Interceptor cohort included a higher relative proportion of AD–/LB– participants, likely due to a patient selection effect. Indeed, while patients in BioFINDER were consecutively recruited through referrals to secondary memory clinics, those in Interceptor underwent multicenter enrollment (19 centers), which more closely reflects the complexity of the real‐world clinical setting and may have favored the inclusion of individuals with a lower probability of having an underlying neurodegenerative disorder.

Parkinsonism, visual hallucinations, and fluctuations—the clinical core features of DLB^28^ investigated—were rare in the Interceptor cohort. No significant differences in the frequency of these features were observed between LB+ and LB– groups, likely due to the small sample size. Although we failed in identifying a clinical profile consistent with the LB+ status, the absolute frequency of attention deficits, motor signs, and psychiatric (i.e., delirium) symptoms was higher in LB+ than in LB– participants. In contrast, the AD+ status was found more frequently in patients with poorer memory and language performance, hippocampal atrophy and hypometabolism of posterior cingulate/precuneus on [18F]fluorodeoxyglucose PET, as well as in those with *APOE* ε4.

In the present study, both the AD+/LB– and AD+/LB+ groups exhibited faster longitudinal cognitive decline than the other groups. In contrast to our finding in the BioFINDER cohort, however, we did not detect a statistically significant difference in longitudinal cognitive trajectories between the AD–/LB– and AD–/LB+ groups. Although the modest LB+ sample size and the limited follow‐up duration likely negatively impacted statistical significance, our data suggest that LB pathology contributes to accelerating longitudinal cognitive decline in AD+ participants, as demonstrated by a steeper CDR increase and a trend toward faster MMSE decline over a 3‐year follow‐up.

Recently, Tosun et al. analyzed AD/LB status and modeled trajectories of longitudinal cognitive assessments over 2 years after CSF sample collection in 1638 individuals from the Alzheimer's Disease Neuroimaging Initiative (ADNI), and also reported no significant differences between AD+/LB+ and AD+/LB– groups.^4^ These divergent results between studies likely depend on the different follow‐up durations, given that in BioFINDER, a considerable number of participants completed a 6‐year follow‐up.

Overall, our findings indicate a notable prevalence of LB pathology but a subtle, often negligible effect on the clinical phenotype at the individual level in the early stages of cognitive decline. These results likely reflect the clinicopathological heterogeneity within the LB+ group, including patients at different stages of LB disease. Indeed, although with reduced sensitivity, αSyn SAA can detect LB pathology even when is limited to the brainstem or amygdala,^29^, ^30^, at a stage in which it may have a relatively minor impact on the MCI phenotype compared to more widespread LB pathology. As a result, identifying prodromal DLB clinically in these patients can be challenging.^31^

Neuropathological studies have extensively documented the contribution of coexisting AD/LB pathologies, and their relative burden, to the clinical phenotype, which is characterized by substantial overlap between DLB and AD features, and a faster disease progression.^32^, ^33^, ^34^ Notably, greater AD pathological burden,^35^, ^36^ as well as female sex,^34^, ^37^ have been associated with a lower number of core DLB clinical features. Similarly, in clinical DLB cohorts, the presence of coexisting AD pathology, as determined by positive CSF amyloid and tau biomarkers, has been associated with more rapid cognitive decline and a higher risk of mortality.^38^, ^39^, ^40^ Taking this evidence into consideration, the greater frequency of female sex, the relatively low frequency of clinical core features of LB disease, and their marginal associations with LB+ status might lead to the conclusion that a significant number of participants with αSyn SAA positivity in our cohort did not have widespread LB pathology. Nonetheless, our longitudinal analysis seems to support the clinical contribution of LB pathology, besides AD pathology, to the progression to dementia. This apparent discrepancy may be attributable to the early disease stage (i.e., MCI), in which the clinical effects of LB pathology may be obscured. In this view, the αSyn SAA adds value to the diagnostic workup of patients with MCI by predicting clinical trajectories and, in clinical trials, assisting patient enrollment and contributing to the assessment of the efficacy of disease‐modifying treatments for AD.

The study is not without limitations, particularly the limited sample size, the low prevalence of AD(±)/LB+ individuals compared to those AD–/LB–, and the relatively short follow‐up duration. Moreover, the lack of systematic assessment of rapid eye movement sleep behavior disorder, a clinical core feature of DLB;^28^ LB‐associated biomarkers; and other supportive DLB features (e.g., smell loss, daytime sleepiness, autonomic disturbance, etc.)^31^ has limited the comprehensive assessment of the effect of LB pathology on the clinical phenotype.

Major strengths include the robustness of our αSyn SAA (validated against the neuropathological gold standard) and our experience with SAA application to large clinical cohorts, as demonstrated by previous studies.

## CONFLICT OF INTEREST STATEMENT

The authors declare no conflicts of interest. Author disclosures are available in the Supporting Information.

## CONSENT STATEMENT

The study was conducted in accordance with the Declaration of Helsinki for the protection of human participants and was approved by the ethics committee of Fondazione Policlinico Universitario Agostino Gemelli (approval code 2251). Additionally, approval was obtained from all local ethics committees of the participating clinical centers. Written informed consent was obtained from participants.

## Supporting information



Supporting Information

Supporting Information
