# Supplementary material for: Prevalence of Lewy body pathology and phenotypic associations in patients with mild cognitive impairment: Evidence from the Interceptor study
Source: Alzheimers Dement (Amst). 2026 Jun 15;18(2):e70382. doi: 10.1002/dad2.70382 (PMC13269012; doi:10.1002/dad2.70382)
Supplement: Supplementary file 2 — Supporting Information [file DAD2-18-e70382-s001.docx]

**Supplementary Table 1**. Missing data in longitudinal analyses (N=323)

| **Variable** | **T0** | **T6** | **T12** | **T18** | **T24** | **T30** | **T36** |
| --- | --- | --- | --- | --- | --- | --- | --- |
| MMSE | 323 (100%) | 305 (94.4%) | 305 (94.4%) | 262 (81.1%) | 226 (70.0%) | 199 (61.6%) | 174 (53.9%) |
| CDR | 321 (99.4%) | 303 (93.8%) | 303  (93.8%) | 258 (79.9%) | 219 (67.8%) | 190 (58.8%) | 167 (51.7%) |

Abbreviations: MMSE = Mini-Mental State Examination, CDR = Clinical Dementia Rating
